# Naringin attenuates cerebral ischemia-reperfusion injury in rats by inhibiting endoplasmic reticulum stress

**DOI:** 10.1515/tnsci-2020-0170

**Published:** 2021-05-13

**Authors:** Li Wang, Zhe Zhang, Haibin Wang

**Affiliations:** Department of Emergency Medicine, The Second Affiliated Hospital, Zhejiang University School of Medicine, Hangzhou 310002, China; Department of Emergency Medicine, The First People’s Hospital of Yuhang District, Hangzhou 311100, China; Department of Radiology, Translational Medicine Research Center, Key Laboratory of Clinical Cancer Pharmacology and Toxicology Research of Zhejiang Province, Affiliated Hangzhou First People’s Hospital, Zhejiang University School of Medicine, Hangzhou 310006, China

**Keywords:** naringin, cerebral ischemia-reperfusion injury, inflammation, oxidative stress, endoplasmic reticulum stress

## Abstract

**Objective:**

This investigation was carried out with an aim of exploring neuroprotection by naringin (Nar) in rats with cerebral ischemia-reperfusion (CI/R) injury and its mechanism.

**Methods:**

Rats were grouped into ischemia-reperfusion (I/R), sham operation (Sham), nimodipine control (NIM), and different doses of Nar (Nar-L, Nar-M, Nar-H) groups. With Zea Longa score for assessment of neurological deficits, dry and wet method for measurement of brain tissue water content, and (2,3,5-triphenyltetrazolium chloride) TTC staining for determination of cerebral infarction volume, the related parameters were obtained and compared. Subsequently, ELISA was introduced to detect levels of proinflammatory cytokines (TNF-α, IL-8) and anti-inflammatory cytokine (IL-10) in the serum as well as superoxide dismutase (SOD) and malondialdehyde (MDA) activities in brain tissue. Western blot was applied to evaluate endoplasmic reticulum stress (ERS)-related proteins expression, including glucose-regulated protein 78 (GRP78), C/EBP homologous protein (CHOP), caspase-12, and activating transcription factor 6 (ATF-6).

**Results:**

Nar significantly alleviated nerve injury and decreased brain tissue water content and brain infraction volume in CI/R injury rats in a concentration-dependent manner. Reduction of TNF-α, IL-8 as well as MDA content and elevation of IL-10 as well as SOD activity were confirmed to be caused by Nar treatment in a concentration-dependent manner. Meanwhile, ERS-related proteins also markedly decreased in the Nar groups.

**Conclusion:**

Nar may achieve neuroprotection and alleviation of CI/R injury by anti-inflammation, anti-oxidation, and inhibiting ERS, and its efficacy is concentration-dependent.

## 1 Introduction

Cerebral ischemia-reperfusion (CI/R) injury, a cerebrovascular disease, occurs as a result of the restoration of blood perfusion after a certain period of cerebral ischemia and hypoxia, which will aggravate the structural and functional damage caused by hypoxia and even lead to more serious complications. CI/R injury-caused severe brain injury and dysfunction is a challenging problem in clinical treatment, and currently no safe and reliable targeted drug was developed for it. Approximately 1 million CI/R injury-caused deaths and 5 million CI/R injury patients who become appear disability or paralysis has worldwide distribution [1]. With high incidence, disability, and mortality, CI/R injury seriously threatens health and life of patients and also leads to a marked reduction in their quality of life [2]. It has been shown that oxidative stress damage indirectly induced by CI/R injury leads to physiological dysfunction of the endoplasmic reticulum, known as endoplasmic reticulum stress (ERS). ERS leads to endoplasmic reticulum protein misfolding, which further induces an unfolded protein response (UPR), while sustained oxidative stress and UPR can activate downstream apoptosis-related pathways and induce apoptosis [3]. And studies have also confirmed that the ERS-mediated apoptotic pathway is thought to play an important role in mediating brain damage after CI/R injury [4]. Therefore, how to effectively inhibit ERS induced by ischemia-reperfusion (I/R) injury will provide new ideas for the treatment of CI/R.

Many studies have found that natural extracts have a preventive effect on CI/R injury [5]. For example, after being treated with sargentodoxa cuneata total phenolic acids, inflammation and oxidative response in brain tissue present to decline, thus relieving CI/R injury in rats [6]; after being treated with total flavonoids of Radix Ilicis Pubescentis, damage of nerve cells in brain tissue and no content was appeared to be reduced, causing improvement in CI/R injury mice [7]. Naringin (Nar), a flavonoid extracted from the grapefruit fruit of the Rutaceae family, can act as free radical scavenger and is an effective anti-oxidation, anti-inflammation, and vascular protection. Many studies have shown that Nar has significant potential in the prevention and treatment of CI/R injury [8]. Nar was found to reduce catalase activity, decrease peroxidation, and significantly improve neurological injury in CI/R injury mice [9]. In addition, Nar could cross the blood-brain barrier and reduce excessive mitophagy, thus preventing CI/R injury [10]. Wang et al. [11] found that Nar reduces oxidative stress and improves mitochondrial dysfunction by activating the Nrf2/ARE signaling pathway in neurons. Okuyama et al. [12] similarly demonstrated that Nar-rich rhizome peel has a neuroprotective effect against CI/R injury in rats.

However, there are fewer studies related to the effect of Nar on ERS and the association with CI/R injury. In view of the previous studies on Nar, we herein investigate the neuroprotection by Nar in CI/R injury rats and the possible ERS-related mechanism of this extract.

## 2 Materials and methods

### 2.1 Preparation of rat model of cerebral ischemia-reperfusion injury

Fifty-four adult Sprague Dawley rats, 27 females and 27 males, weighing 180–220 g, were taken. Random allocation of the rats into 6 groups of 9 was completed, named as sham operation (Sham) group, ischemia/reperfusion (I/R) group, Nimodipine (NIM) group, and Nar low-, median-, and high-dosage (Nar-L, Nar-M, Nar-H) groups. For establishment of I/R injury model, the rats were anesthetized with pentobarbital sodium (40 mg/kg, intraperitoneal injection), and endotracheal intubation was performed while spontaneous breathing was preserved. After that, skin incision was followed to carefully expose the right common carotid artery, internal carotid artery, and external carotid artery. Then in the I/R group, the method described by Zea Longa was applied for blocking internal carotid artery to cause ischemia for 2 h, followed by reperfusion for 24 h. Intragastrical administration of normal saline (1.8–2.2 mL/100 g, once a day) was given 7 days before surgery in the I/R group. In the Sham group, same preoperative treatment and same surgery as the I/R group were done, but with no occlusion using nylon suture. In the NIM group, the surgery was also the same while intragastrical administration of the positive drug nimodipine (15 mg/kg) was given 7 days before surgery. The Nar groups received the same surgery, with intragastrical administration of 25, 50, and 100 mg/kg Nar 7 days before surgery.

### 2.2 Neurological deficit score

After 24 h of perfusion, Zea Longa score [13] was introduced for evaluation of neurological impairment in rats. 0 point: No nerve injury; 1 point: Failure to fully extend forepaw; 2 points: Contralateral circling; 3 points: Contralateral tumbling; 4 points: Failure to walk spontaneously and loss of consciousness.

### 2.3 ELISA

#### 2.3.1 Detection of inflammatory factors in serum

On completion of neurological assessment, 12 h fasting was conducted with the provision of drinking. Next day, their blood was collected for 5 min centrifugation at 500 g/min to take the serum. The final step was the assessment of TNF-α, IL-8, and IL-10 contents in rat serum in keeping with the instructions of the ELISA kit (Abcam, UK) strictly.

#### 2.3.2 Detection of oxidative stress-related parameters in brain tissue

After decapitation, brain tissues removed from 3 rats in each group were ground into homogenates for 10 min centrifugation at 500 g/min. Then the supernatant was taken. The final step was to measure superoxide dismutase (SOD) activity and malondialdehyde (MDA) content in keeping with the instruction of MDA kit and SOD kit (Nanjing Jiancheng Bioengineering Institute, China).

### 2.4 Detection of brain tissue water content by a dry and wet method

On completion of decapitation, brain tissues removed from 3 rats in each group were weighed to obtain the wet weight of the whole brain. The brain tissues were dried in a 105°C oven to a constant weight, which was the dry weight. Bran tissue water content = (wet weight − dry weight)/wet weight × 100%.

### 2.5 TTC staining

On completion of decapitation, brain tissues removed from 3 rats in each group were cut into 5 coronal sections equidistantly. Then after 15 min incubation of these sections immersed in TTC solution, photographs were taken utilizing a digital camera and analysis of images was allowed by using ImageJ verl. 37c NIH software. Based on the determination of cerebral infarction area (pale area) and normal brain tissue area (red area), the percentage of cerebral infarction volume could be calculated.

### 2.6 Western blot

Brain tissues ground into homogenates was used to extract proteins by utilizing RIPA lysis buffer. Then in order to quantify extracted protein concentration, a BCA Kit (Thermo, USA) was applied. Then 25 μg of protein was transported to the PVDF membrane after being separated utilizing SDS-PAGE. On completion of subsequent blocking step by 5% skim milk powder for a period of 2 h, overnight co-incubation of the membrane and glucose-regulated protein 78 (GRP78), C/EBP homologous protein (CHOP), caspase-12, and activating transcription factor 6 (ATF-6) antibodies (Cell Signaling Technology, USA) was performed at 4°C, followed by rinsing step for 3 times utilizing TBST and another 2 h incubation with corresponding secondary antibodies at room temperature. Once the incubation was completed, the sample was washed for 3 times again. Proteins were developed by a chemiluminescent reagent for a period of 3 min. The final step was to obtain the images utilizing a gel imaging system and to analyze protein content utilizing Quantity-One software. Calculation of relative protein expression was allowed with GAPDH as an internal reference.

### 2.7 Statistical analysis

All data were presented as mean ± standard deviation (SD). Statistical analysis was performed using SPSS 22.0. The differences between multiple groups were determined using one-way analysis of variance (ANOVA). Student’s *t*-test analysis was performed to compare the difference between two groups. The criterion of a statistically significant difference was *P* < 0.05.


**Ethical approval:** The research related to animals’ use has been complied with all the relevant national regulations and institutional policies for the care and use of animals.

## 3 Results

### 3.1 Remission of ischemia/reperfusion-induced brain injury by Nar

**Figure 1:**
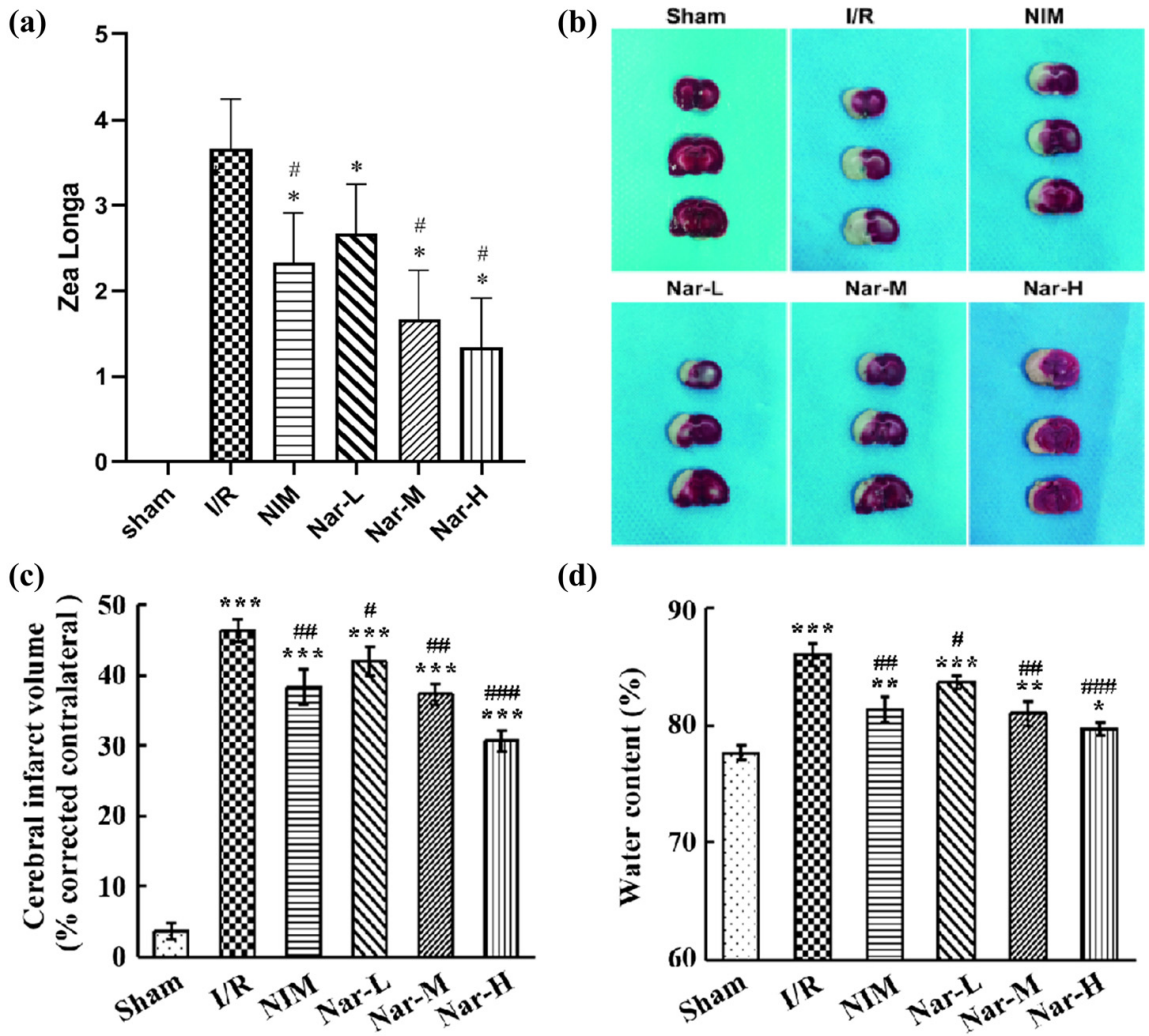
Remission of ischemia/reperfusion-induced brain injury by Nar. (a) Zea Longa score for neurological impairment assessment. (b) Determination of brain tissue infarction utilizing TTC staining. (c) Percentage of brain infarct volume. (d) Assessment of brain tissue water content utilizing dry and wet method. *N* = 3/group. *P < 0.05, **P < 0.01, and ***P < 0.001 vs Sham group; ^#^ *P* < 0.05, ^##^ *P* < 0.01, and ^###^ *P* < 0.001 vs I/R group.

In neurological impairment assessment, rats of the Sham group got 0 point. In comparison with the Sham group, the score representing brain injury severity increased markedly in the I/R group. In comparison with the I/R group, scores decreased markedly in the NIM group and Nar groups (Figure 1a). In the 3 Nar groups, with an escalation of Nar dosage, scores gradually decreased.

In further TTC staining and dry and wet methods, rats of Sham group showed no infarct and relatively low water content in the brain tissue. In comparison with the Sham group, different degrees of infarction and increases of water content could be observed in the other 5 groups (Figure 1b). In comparison with the I/R, nimodipine and Nar could significantly reduce the infarct size and brain water content of rat brain tissue, and Nar was concentration-dependent (Figure 1c and d). Hence, it was evident that Nar was effective in alleviating CI/R injury-induced brain infarction and neurological impairment and in reducing brain tissue water content.

### 3.2 Alleviation of ischemia/reperfusion-caused local inflammation by Nar

**Figure 2:**
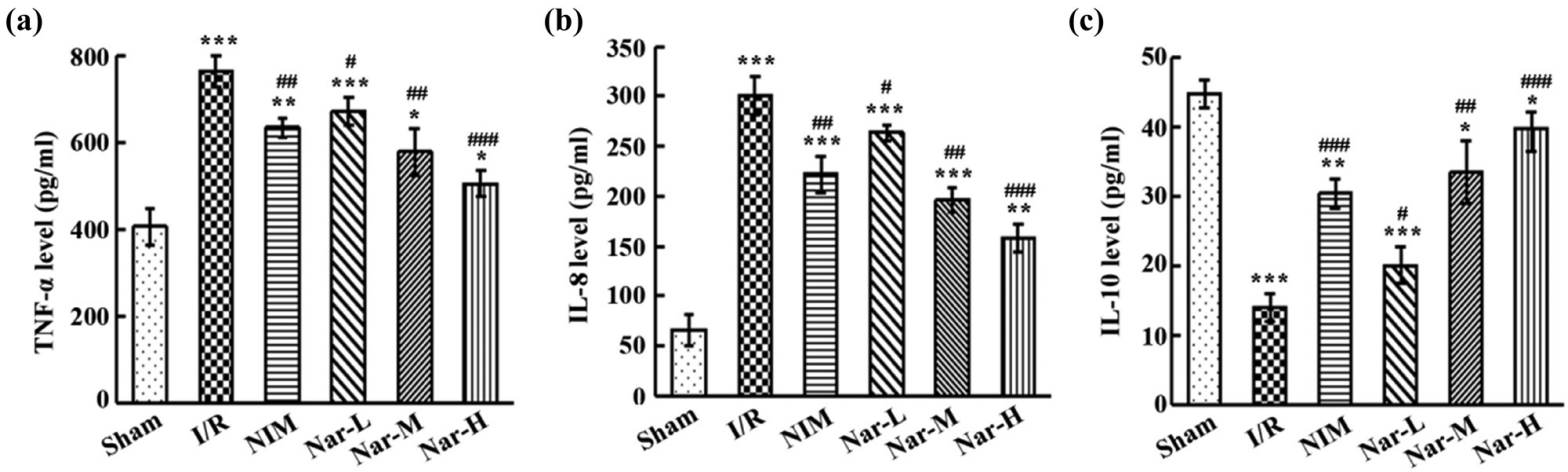
Alleviation of ischemia/reperfusion-caused inflammation by Nar. Evaluation of TNF-α (a), IL-8 (b), and IL-10 (c) levels in the serum of rats. *N* = 3/group. *P < 0.05, **P < 0.01, and ***P < 0.001 vs Sham group; ^#^ *P* < 0.05, ^##^ *P* < 0.01, and ^###^ *P* < 0.001 vs I/R group. This experiment was repeated three times.

From ELISA results, in comparison with the Sham group, serum TNF-α and IL-8 levels in the I/R group raised markedly, while IL-10 level decreased significantly. In comparison with the I/R group, serum TNF-α and IL-8 levels in the NIM group and Nar groups reduced considerably while IL-10 increased significantly, and Nar was concentration-dependent (Figure 2a–c). Hence, it was evident that Nar was effective in alleviating CI/R injury-induced inflammation, and its efficacy was better than that of nimodipine when a certain concentration is reached.

### 3.3 Inhibition of ischemia/reperfusion-caused oxidative stress by Nar

**Figure 3:**
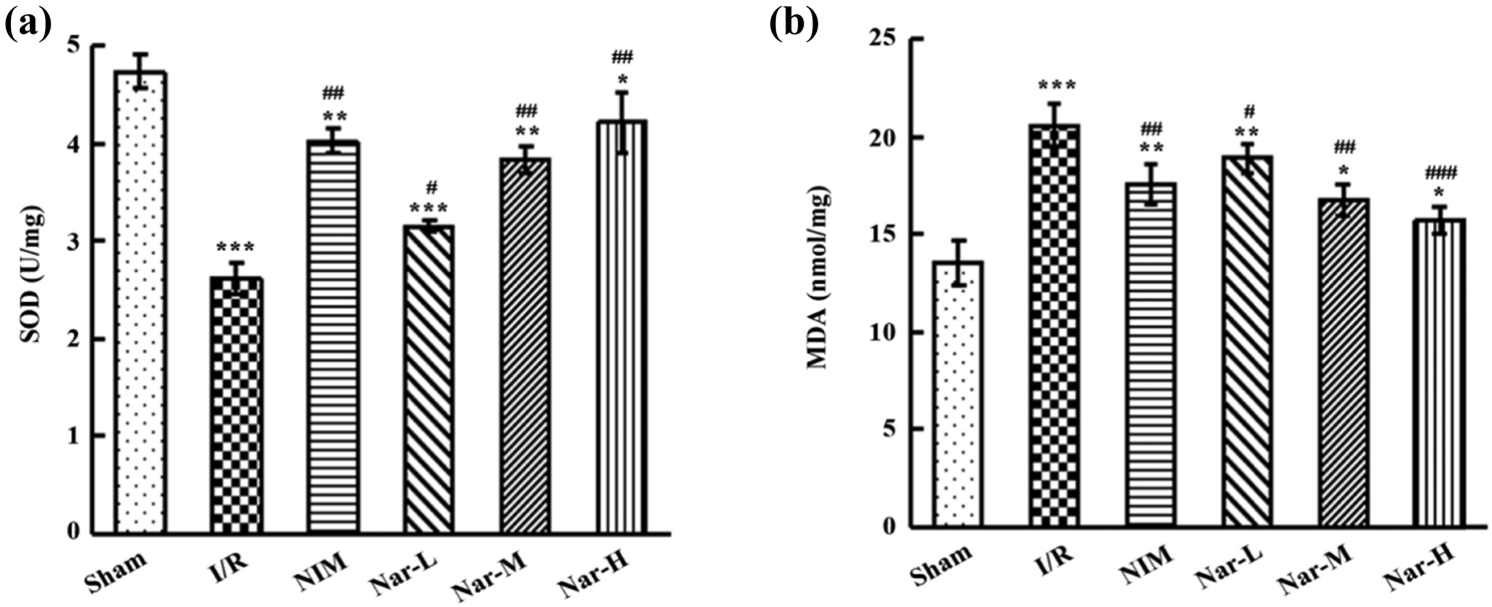
Inhibition of ischemia/reperfusion-caused oxidative stress by Nar. (a) Determination of SOD activity in brain tissue utilizing SOD kit. (b) Assessment of MDA content in brain tissue utilizing MDA kit. *N* = 3/group. *P < 0.05, **P < 0.01, and ***P < 0.001 vs Sham group; ^#^ *P* < 0.05, ^##^ *P* < 0.01, and ^###^ *P* < 0.001 vs I/R group. This experiment was repeated three times.

In comparison with the Sham group, I/R led to a marked reduction of SOD activity and an elevation of MDA content. In comparison with the I/R group, marked decrease of SOD activity and increase of MDA content were revealed in the NIM group and Nar groups (Figure 3a and b). And with the increase of Nar dose, SOD activity was on the rise and MDA content was on the decline, suggesting concentration-dependence of Nar. Compared with nimodipine, high dosage of Nar had better efficacy in reducing oxidative stress. Hence, it was supported that Nar, which functioned in increasing SOD activity and in decreasing MDA activity, could enhance ability to scavenge oxidative free radical, thus causing a reduction of peroxidation to relieve CI/R injury-induced oxidative stress in rat brain tissue. Additionally, with the characteristic of concentration-dependence, Nar in a certain concentration could achieve better efficacy than nimodipine.

### 3.4 Inhibition of ischemia/reperfusion-caused endoplasmic reticulum stress by Nar

**Figure 4:**
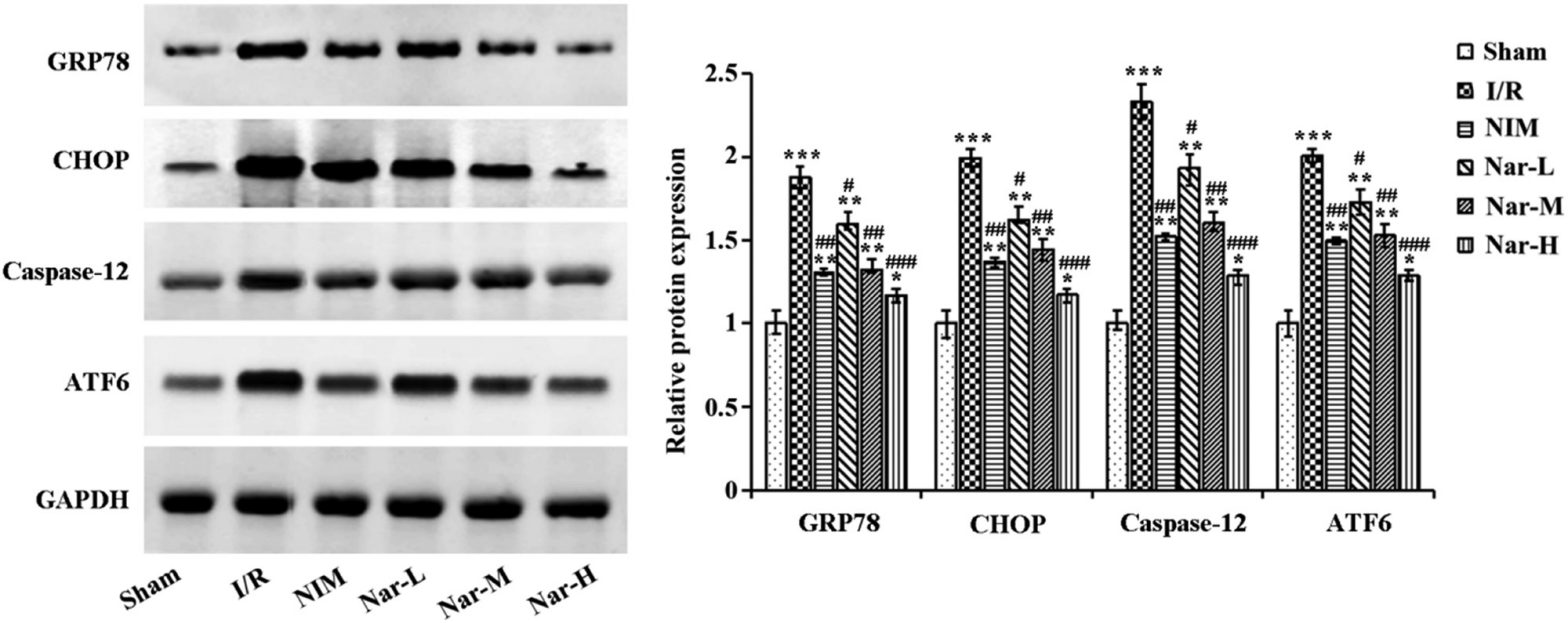
Inhibition of ischemia/reperfusion-caused endoplasmic reticulum stress by Nar. Determination of endoplasmic reticulum stress-related proteins GRP78, CHOP, caspase-12, and ATF6 in the brain tissues utilizing western blot. *N* = 3/group. *P < 0.05, **P < 0.01, and ***P < 0.001 vs Sham group; ^#^ *P* < 0.05, ^##^ *P* < 0.01, and ^###^ *P* < 0.001 vs I/R group. This experiment was repeated three times.

For the purpose of determination of the relationship between the neuroprotective effect of Nar and ERS, ERS-related proteins in the brain tissues were assessed utilizing Western blot, including GRP78, CHOP, Caspase-12, and ATF6. I/R led to a marked increase of the expression of these proteins, but nimodipine and Nar significantly decreased their expression (Figure 4). Nar was concentration-dependent. Collectively, inhibition of CI/R injury-caused ERS by Nar was proved and this extract in a certain concentration could be more effective than nimodipine.

## 4 Discussion

After a period of cerebral ischemia, a significant functional and structural change will occur during blood flow recovery, which is known as CI/R injury. This kind of blood flow recovery can lead to irreversible damage such as brain cell necrosis, cell swelling, and tissue with heterogeneous blood flow [14]. According to a large number of studies on CI/R injury in recent years, CI/R injury has been proved to be a key factor leading to brain injury, and the involvement of neuroinflammation and oxidative stress in the pathophysiological process of CI/R injury has also been confirmed [15].

Based on a rat model of I/R established by suture-occluded method, we first explore the neuroprotection of Nar. The results showed that in comparison with the I/R group, administration of Nar led to higher nerve injury score, decreased infraction volume, and reduced water content in brain tissue, and its efficacy was concentration-dependent. Collectively, the improvement of CI/R injury-caused nerve injury and brain tissue injury by Nar is supported by these outcomes. Previous studies have performed trials which determine that cerebral ischemia is a cause of injury in neuronal cell membranes and glial cells, further resulting in a significant increase in the levels of proinflammatory cytokines TNF-α and IL-8, and their increased levels are related to brain injury severity [16]. Both alleviation of CI/R injury and improvement of stroke prognosis by anti-inflammatory regents and antioxidants have been proved [17,18]. Bai et al. pointed out the reduction of neuroinflammation and enhancement of neurological rehabilitation by IL-10 in rats with spinal cord injury. However, IL-10 level was appeared to be decreased when TNF-α, IL-8, and IL-6 levels increased accompanied by 24 h of middle cerebral artery occlusion [6]. Confirmed by Miao et al. oxidative stress after cerebral ischemia aggravated brain injury, while upregulation of SOD and catalase, which have antioxidant effects, facilitated the recovery of neurons underwent I/R [19]. Previous studies have shown that Nar has a strong antioxidant capacity and the ability to scavenge hydroxyl and superoxide radicals [20,21]. Sugumar et al. also found neuroprotective effects of naringenin against MPTP-induced oxidative stress [22]. For the resolution of the question of how Nar affects CI/R injury-caused inflammation and oxidative stress, ELISA was carried out in our study and found Nar could dose-dependently downregulate TNF-α and IL-8 and MDA, while upregulate IL-10 and SOD. This result means that Nar can enhance ability to inhibit CI/R injury-caused inflammation and to scavenge oxidative free radical, thus causing a reduction of peroxidation to relieve CI/R injury-induced oxidative stress, which is as the previous studies. Collectively, it is speculated that anti-inflammation and anti-oxidative stress of Nar is effective in alleviating neuronal apoptosis, in inhibiting reactive glial cell activation and proliferation, and in improving ischemia-caused neurological impairment.

CI/R injury leads to cell dysfunction, injury or death, and subsequent calcium overload, which triggers subsequent oxidative stress, organelle dysfunction, metabolic disorders as well as inflammation. Further, ischemia, hypoxia, and oxidative stress can induce ERS [23]. And many studies have shown that early administration of natural plant extracts improves CI/R injury. For example, suppression of ERS-mediated neuronal apoptosis by Iridoid glycosides from Radix Scrophulariae was proved, thus reducing local CI/R injury in rats [24]. And downregulation of NF-κB signaling pathway by celastrol was confirmed to cause neuroprotection in ischemic rats [25]. For further clarification of the relationship between the neuroprotection by Nar and ERS, measurement of ERS-related proteins GRP78, CHOP, caspase-12, and ATF6 by western blot was conducted. The results showed that in comparison with the I/R group, Nar group presented a significant reduction of their expression. Among these proteins, ATF6 pathway is activated after ERS [26]. GRP78, a member of the heat shock protein 70 (HSP70) family, is mainly distributed in the ER [27]. Normally, GRP78 binds to the transmembrane proteins PERK, IRE1, and ATF6 and is unable to activate downstream signaling pathways. However, once the UPR is triggered, GRP78 will dissociate from PERK, ATF-6, and IRE1 and bind to the misfolded protein [28]. This in turn induces changes in the ER-mediated apoptotic pathway proteins caspase-12 and CHOP, ultimately inducing apoptosis [29,30]. And several studies have shown that apoptosis advances the pathological process of CIRI [31,32,33]. Tang et al. [34] also demonstrated that Nar inhibited hypoxia/reoxygenation-induced ATF6 expression in H9C2 cells, which in turn inhibited ERS-mediated apoptosis. Thus, in the present study, we hypothesized that Nar could improve CI/R injury by inhibiting ERS.

## 5 Conclusion

Nar may achieve neuroprotection and alleviation of CI/R injury by anti-inflammation, anti-oxidation, and inhibiting ERS, and its efficacy is better than nimodipine after reaching a certain concentration. However, due to the diversity in the efficacy of Nar, its specific molecular mechanism in inhibiting ERS and improving CI/R injury still needs to be verified by subsequent studies, thus ensuring its safety and efficacy in clinical prevention and treatment of CI/R injury.
